# Ouabagenin is a naturally occurring LXR ligand without causing hepatic steatosis as a side effect

**DOI:** 10.1038/s41598-018-20663-z

**Published:** 2018-02-02

**Authors:** Satoru Tamura, Maiko Okada, Shigeaki Kato, Yasuharu Shinoda, Norifumi Shioda, Kohji Fukunaga, Kumiko Ui-Tei, Minoru Ueda

**Affiliations:** 1 Department of Chemistry, Graduate School of Science, Tohoku University, Sendai, Miyagi 980-8578 Japan; 2 Institute of Medical Science, St. Marianna University Graduate School of Medicine, Kawasaki, Kanagawa 970-8551 Japan; 3 Iwaki Meisei University, Iwaki, Fukushima, 970-8551 Japan; 4 Research Institute of Innovative Medicine, Tokiwa Foundation, Iwaki, Fukushima, 972-8322 Japan; 5 Graduate School of Pharmaceutical Sciences, Tohoku University, Sendai, Miyagi 980-8578 Japan; 6 Graduate School of Science, The University of Tokyo, Tokyo, 113-0032 Japan; 7 Present Address: School of Pharmacy, Iwate Medical University, Shiwa-gun, Iwate, 028-3694 Japan; 8 Present Address: Genome regulation and Molecular Pharmacogenomics, School of Bioscience and Biotechnology, Tokyo University of Technology, Hachioji, Tokyo, 192-0982 Japan

## Abstract

Ouabagenin (OBG) is an aglycone of the cardiotonic steroid ouabain and until now was considered a biologically inactive biosynthetic precursor. Herein, we revealed that OBG functions as a novel class of ligand for the liver X receptor (LXR). Luciferase reporter assays and *in silico* docking studies suggested that OBG has LXR-selective agonistic activity. In addition, OBG repressed the expression of epithelial sodium channel (ENaC), a LXR target gene, without causing hepatic steatosis, a typical side effect of conventional LXR ligands. This remarkable biological activity can be attributed to a unique mode of action; the LXR agonist activity mainly proceeds through the LXRβ subtype without affecting LXRα, unlike conventional LXR ligands. Thus, OBG is a novel class of LXR ligand that does not cause severe side effects, with potential for use as an antihypertensive diuretic or a tool compound for exploring LXR subtype-specific biological functions.

## Introduction

Ouabain (OUA) is a cardiac glycoside natural product discovered in the seeds of plants belonging to the genus *Strophanthus*. OUA has been used as a therapeutic agent to treat cardiac insufficiency. Recently, OUA has attracted attention as an endogenous digitalis-like factor that controls blood pressure in mammals^1,2^. The cardiotonic activity of OUA can be accounted for by its ability to inhibit Na^+^/K^+^-ATPases following Ca^2+^ influx in myocardium cells and vascular endothelial cells^3,4^. By contrast, no biological function has been reported for the aglycone precursor ouabagenin (OBG) since its isolation from *Strophanthus hispidus* in 1935, although it does bind Na^+^/K^+^-ATPases with a much lower affinity than OUA^5,6^. OBG is considered a biologically inactive biosynthetic precursor of OUA. However, OBG belongs to a unique class of highly oxidised sterols called cardenolides, and it would not be surprising if OBG possesses unknown biological functions because recent studies on oxysterols revealed novel activities in mammals, such as the induction of inflammation^7^, promotion of mitochondrial impairment^8,9^, stimulation of osteoclast precursor migration^10^ and attenuation of hepatic steatogenesis^11^. Based on their structures, OUA and OBG belong to a unique class of oxysterols that contain an A/B/C/D-ring system with six hydroxyl groups. Endogenous oxysterols serve as ligands for liver X receptors (LXRs), which belong to the steroid nuclear receptor (NR) superfamily, and OBG could conceivably serve as a NR ligand.

NRs are DNA-binding transcriptional factors that have been associated with a wide variety of biological and pathological processes^12^. A subgroup of NRs exhibit ligand-dependent gene regulatory functions, but ligand-dependent functions have not been demonstrated for ‘Orphan’ NRs, and cognate endogenous ligands have not been identified. Many aspects of NR-mediated transcriptional control have been described for both gene activation and gene repression. Furthermore, different naturally occurring and synthetic ligands can bind to the same NR to elicit diverse outcomes^13–16^. This ligand-induced specificity is generally understood to be due to differences in ligand binding-induced structural alterations in the ligand-binding domain (LBD) located in the C-terminal E domain of NR proteins. Since the E domain and N-terminal A/B domains are docking sites for transcriptional co-regulators^17^, these ligand-induced structural alterations may be transmitted through the whole NR structure, thereby generating a variety of interfaces for NR- co-regulator association. NRs act as transcriptional co-regulators in both ligand-bound and free states, co-regulating transcriptional processes, and chromatin modifications and reorganisation. The combinations of NRs and co-regulators are believed to determine the specific targeting of sets of genes^18^.

The present study showed that OBG belongs to a novel class of naturally occurring LXR ligands that do not cause the unwanted side effects observed with conventional LXR ligands. OBG is an agonist of LXRα and LXRβ in *in vitro* transactivation assays. However, in a mouse animal model, unlike the conventional LXR agonist T0901317 in transcriptional activation experiments^19–21^, OBG did not induce hepatic steatosis following activation of LXR-targeted genes, even though it was equally effective at repressing the epithelial sodium channel (ENaC) in mouse kidney^22^. OBG was shown to act almost exclusively through LXRβ in a renal cell line, hence the avoidance of LXRα-related side effects. These results suggest that OBG may be a potent antihypertensive diuretic drug lead not associated with undesirable side effects.

## Results

### OBG serves as a LXR ligand

**Figure 1:**
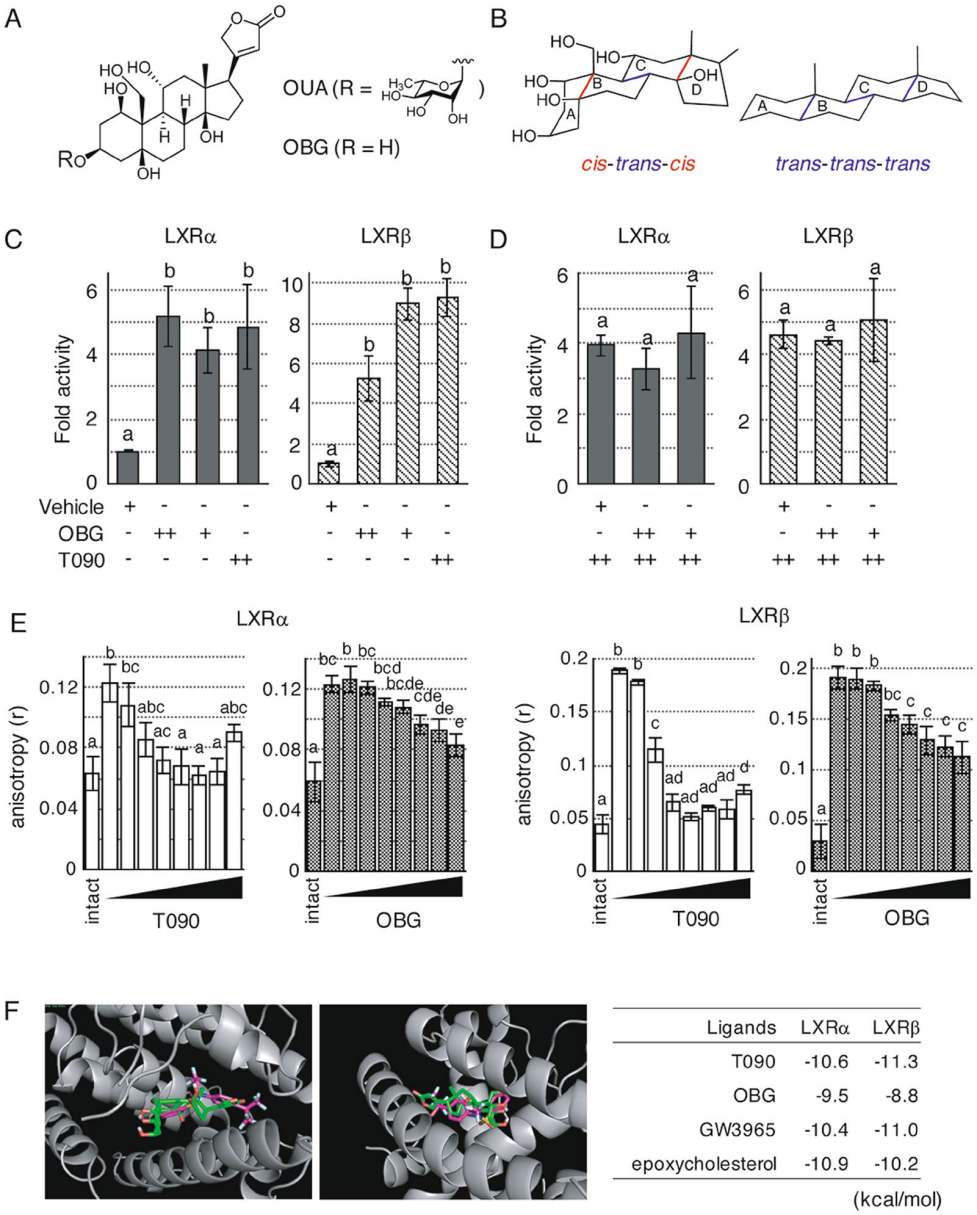
Identification of OBG as a ligand for LXRs. (**A**) Chemical structure of OBG and ouabain (OUA). (**B**) OBG has a unique steroidal ring fusion skeleton. OBG is built around a *cis*-*trans*-*cis*-fused A/B/C/D-ring system (left panel), whereas most normal steroid hormones have an *all*-*trans*-fused ring system (right panel). (**C** and **D**) OBG displays agonistic activity towards LXRs in luciferase reporter assays. 293 T cells transfected with LXRs/RXR, the DR4-tk-Luc reporter and the Renilla reporter plasmid (as a control) were subjected to luciferase assays at 48 h after treatment with 10^−8^ M or 10^−9^ M OBG and/or 10^−9^ M T0901317. The fold change in transcriptional activity is expressed as the mean ± SD of triplicate experiments. Values followed by different letters are statistically different according to analysis of variance (ANOVA) followed by SNK tests (P < 0.01). (**E**) Competitive binding assay using the fluorescent LXR ligand by detection of fluorescent anisotropy. To the solution of complex of LXR-LBD (0.5 μM) and the fluorescent LXR ligand (0.5 μM), OBG or T0901317 was added with the concentration of 0 μM, 0.1 μM, 0.3 μM, 1.0 μM, 3.0 μM, 10 μM, 30 μM, or 100 μM. Intact means fluorescent anisotropy of the fluorescent ligand in the buffer without protein. Bar graphs represent means ± SD of dependent three experiments and values followed by different letters are statistically different according to ANOVA followed by SNK tests (P < 0.01). (**F**) OBG has a strong affinity for LXRs in *in silico* docking simulations. Using previously reported datasets from crystal structures of the LXR/T0901317 complex, OBG was subjected to *in silico* docking simulations and the results were compared with other known LXR ligands. After docking simulation, both superimposed structures between OBG (green colored structure) and T0901317 (magenta colored structure) were settled down around the similar pocket of LXRα (gray colored ribbon in left panel) and LXRβ (gray colored ribbon in right panel). Based on the simulations, the stabilisation energy was calculated (right table). T0901317 and GW3965 are synthetic ligands of LXRs, and 24(*S*), 25-epoxycholesterol is a natural ligand.

The structure of OBG places it into a unique class of oxysterols with six hydroxy groups and a unique *cis*-*trans*-*cis*-fused A/B/C/D-ring system (Fig. 1A,B, left panel), distinct from *all-trans*-fused A/B/C/D-ring systems of other steroid hormones such as progesterone or androsterone (Fig. 1B, right panel). Many steroids are known to function as endogenous ligands of NRs. Due to the bent steroid structure arising from the *cis*-*trans*-*cis* ring in OBG, we wondered whether OBG serves as a NR ligand with the potential to induce structural alterations in OBG-bound NRs, thereby influencing their bioactivity. To screen potential NR targets, we assessed the agonistic/antagonistic effects of OBG for NR-mediated transactivation using the dual luciferase reporter assay system^11^. LXR/retinoid X receptor (RXR) expression plasmids, a luciferase reporter plasmid for LXRs (DR4-tk-luciferase reporter; DR4-tk-Luc), and a pCMV-Renilla luciferase reporter (Renilla-CMV-Luc) plasmid, were co-transfected into 293 T cells, and luciferase assays were performed with OBG or with the conventional LXR ligand, T0901317. The results revealed no agonistic/antagonistic activity against the nine NRs tested (farnesoid X receptor [FXR], vitamin D receptor [VDR] and five steroid receptors) with OBG up to a concentration of 10^−6^ M (Supplementary Fig. S1). However, OBG was found to exert an agonistic effect on the transactivation function of both LXRα and LXRβ at 10^−8^ and 10^−9^ M, which makes it as effective as the widely used synthetic LXR agonist T0901317 (Fig. 1C)^21^. Moreover, no antagonistic effect was observed at the same concentration (Fig. 1D). These results suggest that OBG serves as a potential agonist for LXRs^23^. Additionally, OUA was also used in the same reporter assay for LXR, but the high cytotoxicity of OUA prevented the normal function of the assay system. Subsequent MTT assays to measure the cytotoxicity of OBG and OUA against 293 T cells showed that the cytotoxicity of OUA was 1000-fold higher (IC_50_ = 11.5 μM for OBG and 6.3 nM for OUA). OBG can therefore be considered as a LXR ligand that avoids the cytotoxicity associated with OUA.

Next, we tried to ensure the direct binding between LXR and OBG by competitive assay. The increase of fluorescent anisotropy of the known fluorescent LXR ligand (Supplementary Fig. S2) incubated with LXR has been reported^24^. Namely, if OBG would bind to LXR competitively with the fluorescent ligand, the decrease of fluorescent anisotropy of the fluorescent ligand with LXR by addition of OBG would be observed. As shown in Fig. 1E, the fluorescent LXR ligand incubated with LXRα and β showed about 0.12 and 0.19 of fluorescent anisotropy, respectively. Addition of OBG or T0901317 to the solution of this complex lead significant decrease of fluorescent polarization (Fig. 1E). The results suggested OBG bound LXR competitively against the fluorescent LXR ligand and OBG worked as a ligand of both LXRα and β which was consistent with the result of reporter assay.

To further characterise OBG as a LXR ligand, its binding affinity for NRs was evaluated by *in silico* docking and compared with its cognate ligand (Fig. 1F). The reported crystal structure of the LXR-T0901317 complex^25,26^ was used as a template, and T0901317 was replaced into a ligand of which structure was minimised beforehand. As a result, OBG was located in a position similar to that of T0901317 in both LXRα- (Fig. 1F, left panel) and LXRβ-binding pockets (center panel). In addition, calculation of OBG-LXR complexes indicated similar stabilisation energies for the cognate synthetic ligands T0901317 and GW3965, and the natural ligand, 24(*S*),25-epoxycholesterol^21,27–29^ (Fig. 1F, right panel). By contrast, simulated complexes of OBG with various NRs including the steroid receptors MR, GR and AR had a much higher stabilisation energy than the NR-original ligand complex (Supplementary Table S1). In particular, although VDR and FXR are structurally and functionally close to LXR, the calculated stabilisation energy was so high that OBG appears to be readily displaced from VDR or FXR (Supplementary Fig. S3, Supplementary Table S2). Taken together, these results suggest that OBG is a selective ligand for LXRs that binds directly.

### OBG binds LXR without inducing hepatic steatosis

**Figure 2:**
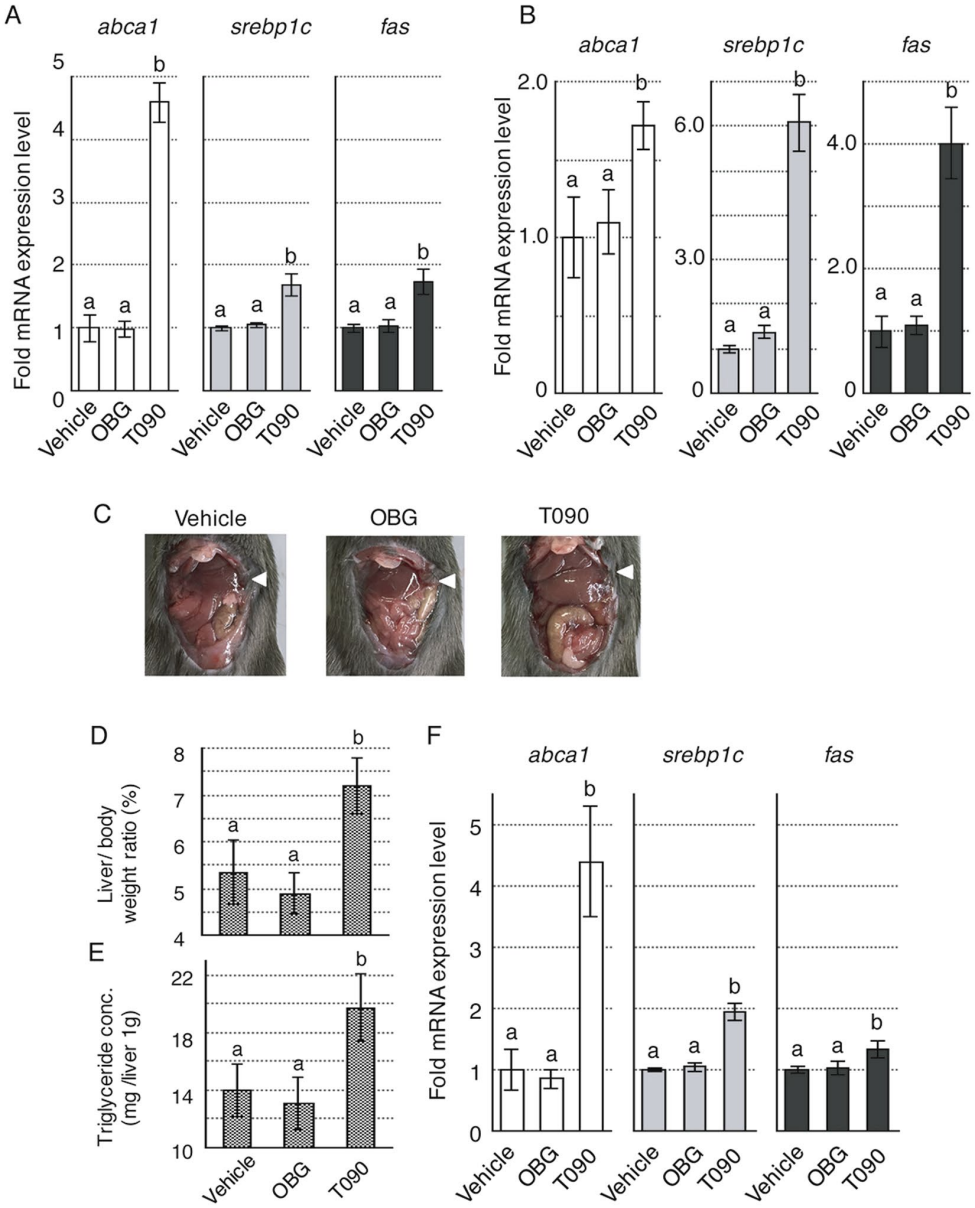
OBG did not cause hepatic steatosis. (**A**,**B**) OBG does not up-regulate the mRNA levels of lipogenesis-related genes in the liver. The mRNA levels of *abca1*, *srebp1c* and *fas* in Hepa1–6 cells (**A**) or HepG2 cells (**B**) were measured at 48 h after treatment with 10^−6^ M OBG or 10^−6^ M T0901317 by qRT-PCR and normalised against *gapdh*. The fold change in vehicle versus ligand-treated cells is shown. Means ± SD are calculated from triplicate measurements in three independent experiments. Values followed by different letters are statistically different based on ANOVA followed by SNK tests (P < 0.01). (**C**–**E**) OBG treatment does not cause the undesirable symptoms of hypertrophic liver. Representative photographs of mouse liver samples after 10 mg/kg OBG or T0901317 treatment are shown. Each liver was indicated by arrow (**C**). Ratio of liver to body weight (**D**) and the triglyceride concentration in the liver (**E**) are depicted. Means ± SD were calculated from biological replicates (vehicle, n = 5; OBG, n = 4; T0901317, n = 4). Values followed by different letters are statistically different based on ANOVA followed by SNK tests (P < 0.01). (**F**) Effect of OBG on lipogenesis-related gene expression in M-1 cells. Levels of *abca1*, *srebp1c* and *fas* mRNA were determined as described in Fig. 2A.

LXR mainly functions in the liver and participates in the regulation of cholesterol, fatty acid and bile acid metabolism, as well as glucose homeostasis^20,30–35^. Two LXR subtypes exhibit different tissue distributions; while LXRβ is distributed ubiquitously, LXRα is highly expressed in metabolically active tissues such as liver. Activation of LXRα triggers fatty liver disease and up-regulates genes associated with lipid metabolism, such as those encoding sterol regulatory element-binding protein-1c (SREBP1c), ATP-binding cassette transporter A1 (ABCA1) and fatty acid synthase (FAS), which are believed to cause hepatic lipogenesis^19–21,36,37^. Next, we evaluated the mRNA expression levels of *srebp1c*, *abca1* and *fas* in the Hepa1–6 murine hepatocellular carcinoma cell line (Fig. 2A) or HepG2 human liver cancer cell line (Fig. 2B). The results showed no up-regulation following addition of OBG at 1 μM, whereas significant up-regulation was observed with T0901317 in both of Hepa106 and HepG2. These results confirmed OBG as a LXR ligand that does not induce fatty liver disease.

To assess the potential of OBG as a tissue-specific LXR ligand, its effects on hepatic lipogenesis were compared with T0901317 using conditions described in a previous report^21^. Male C57BL/6 mice were treated with each ligand once per day at a dose of 10 mg/kg for 5 days, and liver weight and hepatic triglyceride content were measured on day 6. During the experiment, we observed no apparent difference in dietary bulk or the amount of locomotion activity among the three mice groups (controls, OBG-treated and T0901317-treated). Moreover, any obvious side effects such as diarrhoea, inflammation or weight loss weren’t observed (data not shown). Surprisingly, as shown in Fig. 2C, no significant change was observed in OBG-treated mouse liver tissue; the liver/body weight ratio of OBG-treated mice (4.89% ± 0.45%) was almost the same as that of controls (5.35% ± 0.67%), whereas a significant increase was observed with T0901317-treated mice (7.19% ± 0.60%; Fig. 2D). Additionally, T0901317 treatment caused hypertrophic liver symptoms that were not observed in OBG-treated mice. These symptoms could be attributed to an increase in the triglyceride content in the liver because T0901317 caused hepatic lipogenesis^36^. Treatment with T0901317 leads to the accumulation of significantly higher concentrations of triglycerides (20.24 ± 1.08 mg/g liver) in the liver compared with controls (15.41 ± 1.77 mg/g liver), but the concentration of triglycerides with OBG (13.43 ± 2.25 mg/g liver) was similar to controls (Fig. 2E). These results demonstrated that OBG is a unique LXR ligand that does not cause fatty liver disease in mice.

This peculiar property of OBG as a LXR ligand might be due to decomposition of OBG in the liver, since sterols are typically metabolised in this organ. We therefore evaluated expression of genes related to lipogenesis in a kidney cell line after treatment with LXR ligands. However, as displayed in Fig. 2F, expression of *srebp1c*, *abca1* and *fas* following exposure to OBG and T0901317 in the M-1 cells, derived from the collecting duct in mouse kidney, was similar to that in Hepa1–6 cells. Thus, the liver does not appear to diminish the ability of OBG to bind LXR. Furthermore, up-regulation of lipogenesis-related genes except *srebplc* by T0901317 was cancelled under the LXRα knock-down condition whereas three were consistently up-regulated by T0901317 under LXRβ knock-down condition. OBG never up-regulated these lipogenesis-related genes under the both of conditions (Supplementary Fig. S4). These genes were reported to be controlled mainly by LXRα in the liver^19–21,36,37^, so in kidney *abca1* and *fas* may also be regulated by similar mechanism mainly through LXRα but *srebplc* may be regulated by other system in kidney.

### OBG is a unique LXR ligand

**Figure 3:**
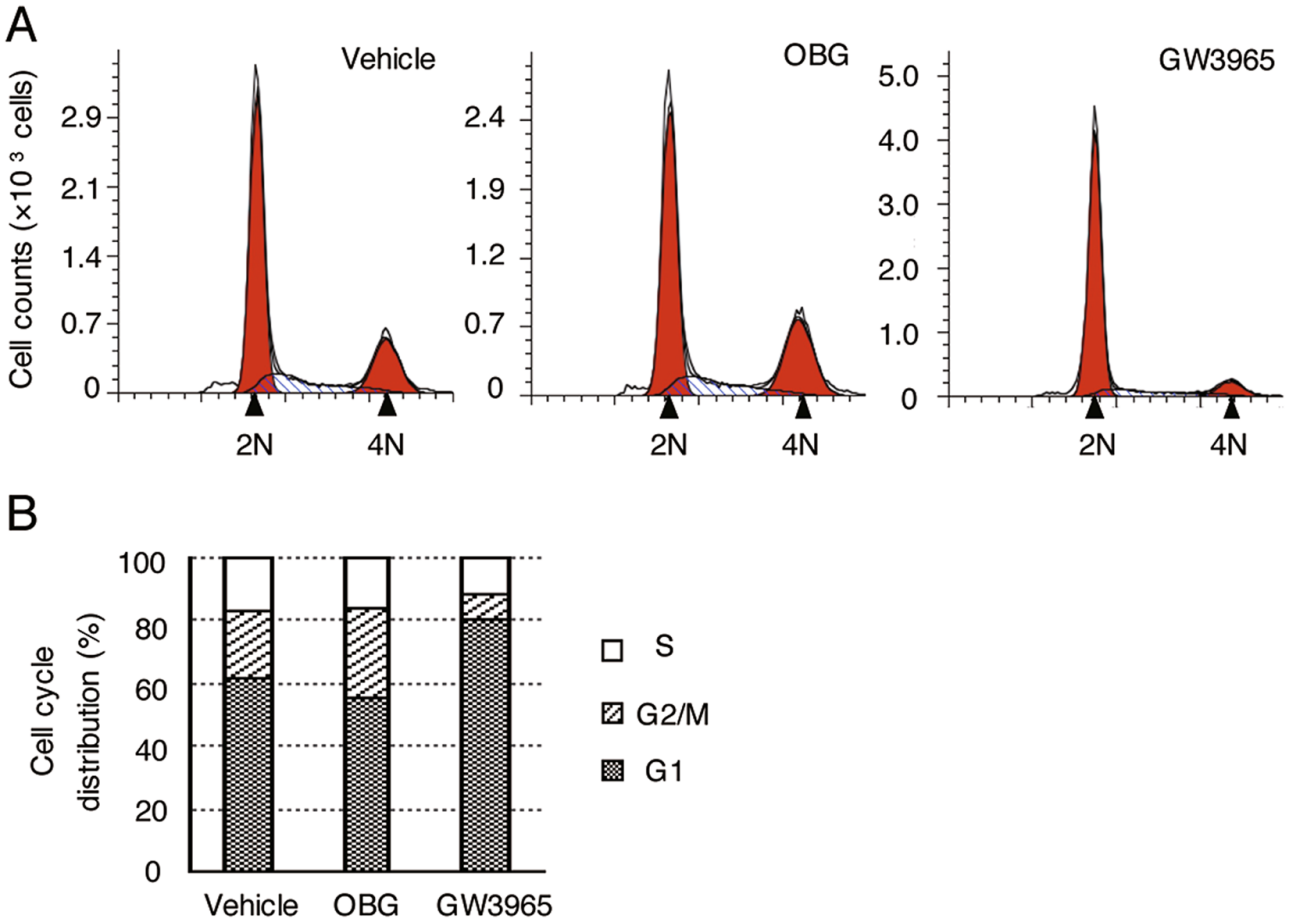
OBG had minimal effect on the cell cycle in HCT116 cells. (**A** and **B**) HCT116 cells treated with 5 × 10^−5^ M OBG or GW3965 were subjected to flow cytometry using propidium iodide. Cell cycle distributions are shown as histograms (**A**). The ratio of each stage was analysed by Modi-Fit (**B**).

In addition to not inducing hepatic lipogenesis, OBG demonstrated other differences from conventional LXR agonists T0901317 and GW3965. T0901317 is a ligand for FXR as well as LXR, and disrupts the expression of apolipoproteins^38^ and fatty acid metabolism^39^. Reporter assays revealed that OBG is not an agonist for FXR, despite T0901317 exhibiting agonistic activity for FXR at 1 μM (Supplementary Fig. S1). We therefore presumed a higher LXR selectivity for OBG than for T0901317. To further investigate its bioactivity as a novel LXR ligand, we examined the toxicity of OBG as a side effect. Cytotoxicity against M-1 cells was tested, and OBG caused minimal inhibition of cell proliferation, even at 0.1 mM, whereas T0901317 and GW3965 showed high cytotoxicity, with IC_50_ values of 12 μM and 3.2 μM, respectively (Supplementary Fig. S5). Furthermore, GW3965 causes cell cycle arrest at the G1 stage in HCT116 colorectal cancer cells^40^. This was tested in the present study, and OBG barely affected the cell cycle stage in HCT116 cells (Fig. 3), whereas GW3965 induced robust G1 cell cycle arrest as reported previously.

Thus, OBG displayed unexpected biological activities despite its LXR agonistic activity and similarity to conventional LXR ligands T0901317 and GW3965, both in the reporter assay and the docking simulation.

### OBG induces down-regulation of ENaC in a LXRβ-specific manner

Recent studies revealed novel functions for LXR ligands in kidney, implicating them as potential blood pressure regulators^41,42^. In addition, synthetic LXR agonists such as T0901317 and GW3965 are reported to decrease sodium transport in collecting duct cells by lowering the expression of ENaC in the cell membrane^22^. ENaC is required for sodium reabsorption in the kidney, which is essential for the maintenance of sodium and water homeostasis, and hence blood pressure stability^43,44^. However, the molecular basis by which LXR ligands, especially naturally occurring ligands, exert their biological function, remains to be elucidated.

**Figure 4:**
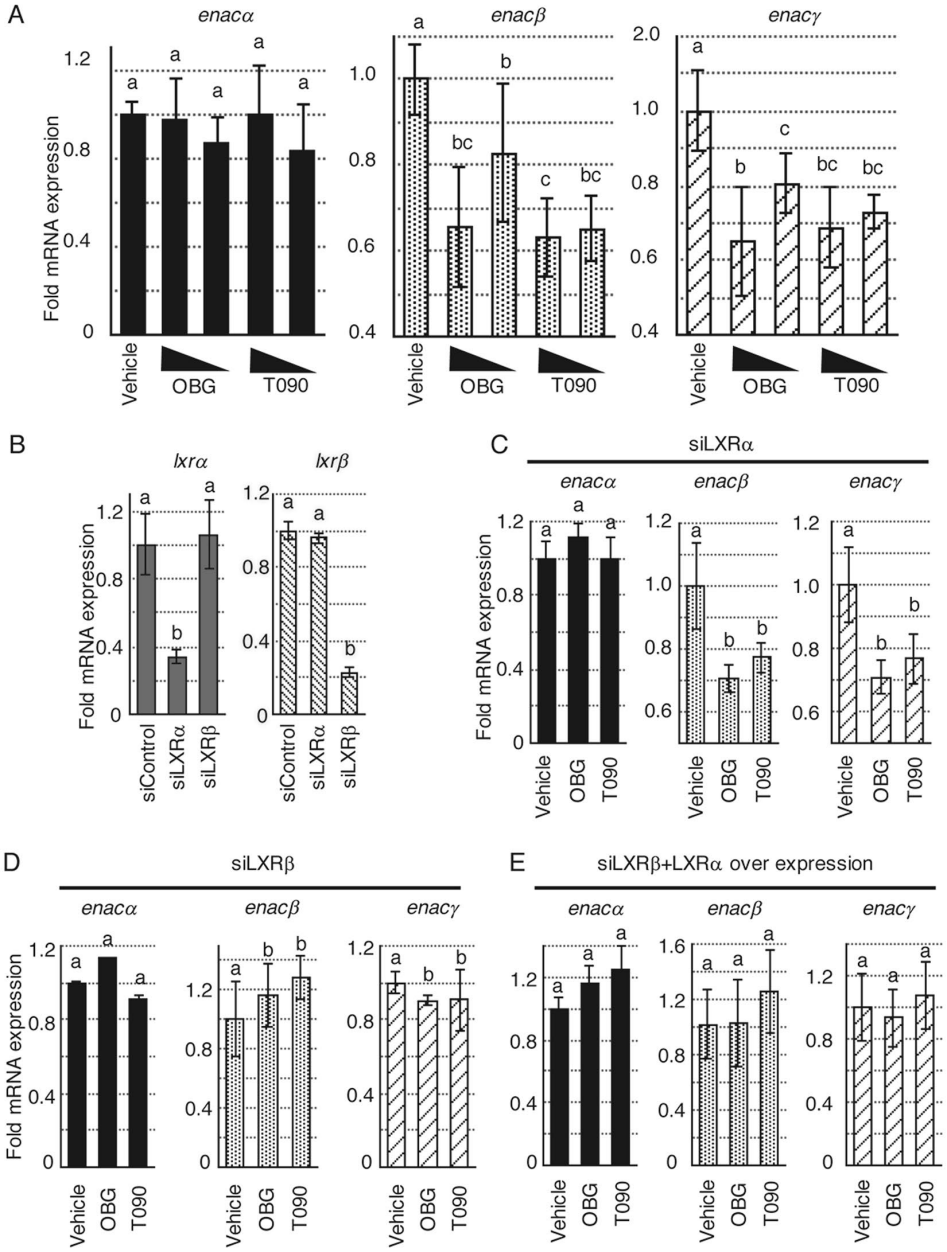
OBG repressed *enac* mRNA expression levels in a LXRβ subtype-specific manner in murine collecting duct M-1 cells. (**A**) OBG repressed the mRNA expressions of *enac*s. The ligand concentration was 10^−6^ M or 10^−7^ M. mRNA expression levels of *enac-*α, *enac-*β and *enac-*γ in M-1 cells were determined as described in Fig. 2A. The amount of mRNA was normalised against *gapdh*, then compared with vehicle. Bar graphs represent means ± SD, and values followed by different letters are statistically different according to ANOVA followed by SNK tests (P < 0.01). (**B**) LXR subtype-selective knockdown in M-1 cells transfected with corresponding siRNAs was confirmed by qRT-PCR. The amount of mRNA was normalised against *gapdh*, then compared with siControl. (**C** and **D**) LXRβ but not LXRα mediates the regulation of ENaC by OBG. Under LXRα (**C**) or LXRβ (**D**) knockdown conditions, mRNA levels of each *enac* subunit in M-1 cells treated with 10^−6^ M ligand were analysed as described in Fig. 4A. (**E**) Overexpression of LXRα is unable to rescue the loss of OBG-induced *enac* repression by siLXRβ. mRNA expression levels of *enac* in M-1 cells transfected with both LXRα expression plasmid and siRNA for LXRβ were analysed as described in Fig. 4A.

To probe the influence of OBG/LXR signalling, we tested the involvement of ENaC regulation signalling in kidney. Firstly, the effects of OBG on the ENaC transcription were examined in M-1 cells. ENaC functions as a complex composed of α, β and γ subunits in rodents^45–48^. Extracted mRNA from M-1 cells treated with OBG or conventional LXR ligands for 24 h was subjected to real-time quantitative reverse transcription PCR (qRT-PCR) using each specific primer corresponding for *enac-*α, *-*β *and -*γ. A decrease in mRNA expression levels of *enac-*β *and -*γ by conventional synthetic ligands for LXR was observed as previously reported^22^. However, *enac-*α transcription was not affected by these compounds in M-1 cells (Fig. 4A and Supplementary Fig. S6). OBG also down-regulated transcription of the *enac*s gene, indicating comparable bioactivity of conventional LXR ligands.

Next, we assessed the dependency of LXR-mediated ENaC regulation on these ligands. LXR exists in two subtypes, LXRα and β, both activated by the same ligands, presumably due to the high sequence and structural similarity. As shown in Fig. 4B, the efficiency of LXR subtype-selective knockdown was confirmed by qRT-PCR, and the depletion of each subtype was tested (Fig. 4B). Depletion of *lxr*β completely cancelled the OBG-induced repression of *enac* expression (Fig. 4D), while depletion of *lxr*α did not affect the expression of any of the *enac* subunits (Fig. 4C). In addition, the expected suppression of *enac*s was observed with T0901317, and this was LXRβ-dependent. However, *lxr*α mRNA levels in M-1 cells were about one hundredth of the *lxr*β mRNA levels (Supplementary Fig. S7). Thus, the role of LXRα was further assessed in LXRβ knockdown and LXRα overexpressing M-1 cells. As shown in Supplementary Fig. S8, both LXRβ knockdown and LXRα overexpression were confirmed, and cells were then treated with OBG or T0901317. Neither OBG nor T0901317 couldn’t restore the down-regulation of *enac*s, even though LXRα was overexpressed. (Fig. 4E). Thus, these results indicated that the suppressive effects of LXR ligands on ENaC expression were exerted in a LXRβ-specific manner in M-1 cells.

**Figure 5:**
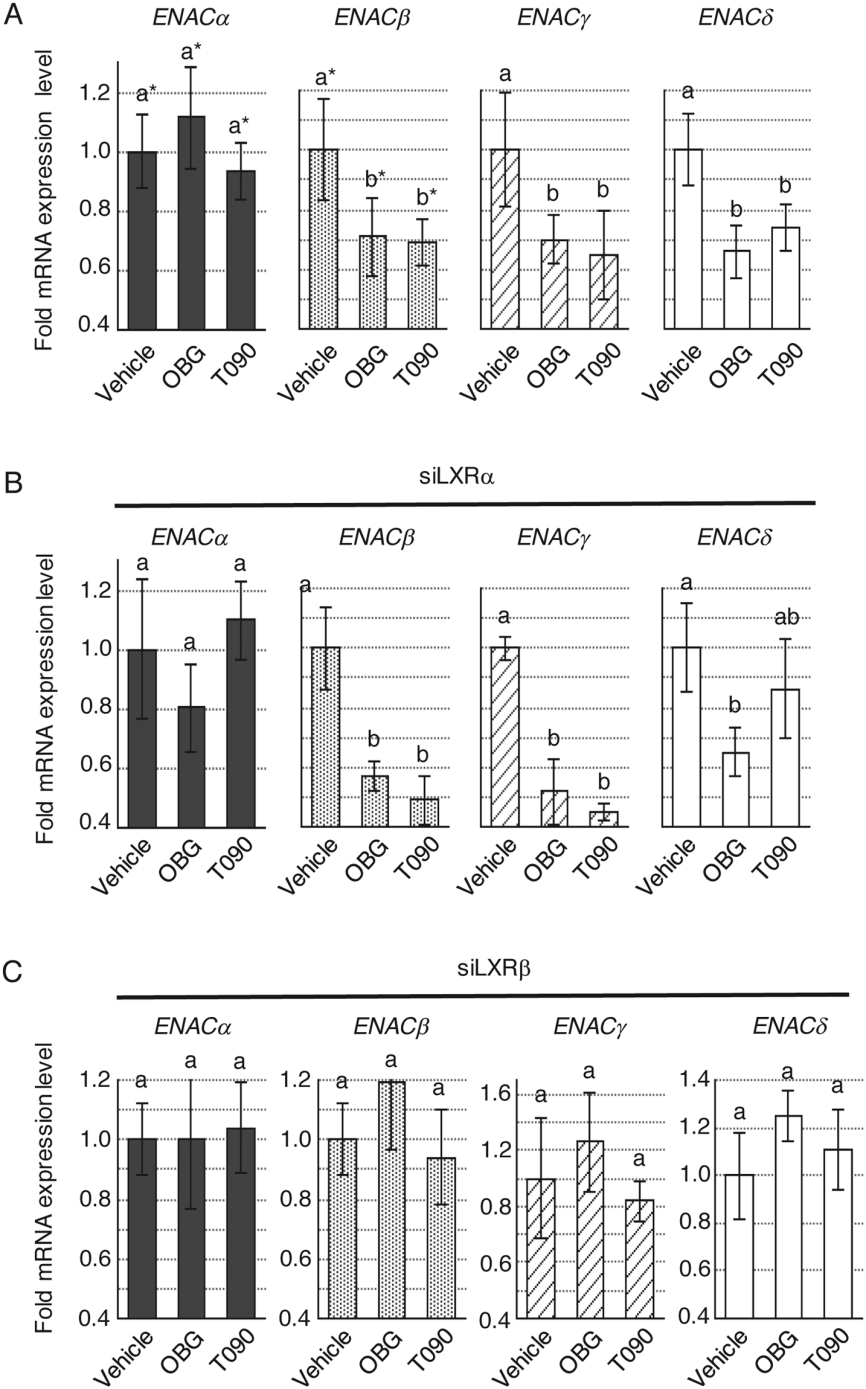
OBG-repressive effects for *ENAC*s were conserved in human embryonic kidney 293 T cells. (**A**) OBG repressed the mRNA expressions of *ENAC*s. The concentration of ligand was 10^−6^ M. mRNA expression levels of *ENAC-*α, *ENAC-*β, *ENAC-*γ and *ENAC-*δ in 293 T cells were determined as described in Fig. 2A. The amount of mRNA was normalised against *GAPDH*, then compared with vehicle. Bar graphs represent means ± SD, and values followed by different letters are statistically different according to ANOVA followed by SNK tests (P < 0.01).(**B** and**C**) LXRβ but not LXRα mediates the regulation of *ENAC*s by OBG. Under LXRα (**B**) or LXRβ (**C**) knockdown conditions, mRNA levels of each *ENAC* subunit in 293 T cells treated with 10^−6^ M ligand were analysed as described in Fig. 5A.

Moreover, the similar repressive functions of LXR ligands on *ENA*C expression were also observed in human embryonic kidney 293 T cell line. OBG and T0901317 reduced the expression level of *ENACs* except ENACα as shown in Fig. 5A. *ENAC*δ, a gene for epithelial sodium channel subunit found in human not in rodent. These results suggested that repressive effects for ENACs were conserved in human cell lines. Considering the almost equal expression levels of LXRα and β, the repressive effects of LXR ligands on *ENAC*s *except ENAC*δ were in an LXRβ-specific manner (Fig. 5B and C, Supplementary Figs S9 and S10). Only in the *ENAC*δ regulation, T0901317 repress it through both of LXRα and LXRβ, which suggest there are different regulation mechanisms for *ENAC*δ in T0901317 and OBG.

### OBG down-regulates ENaC mRNA expression in mouse kidney

**Figure 6:**
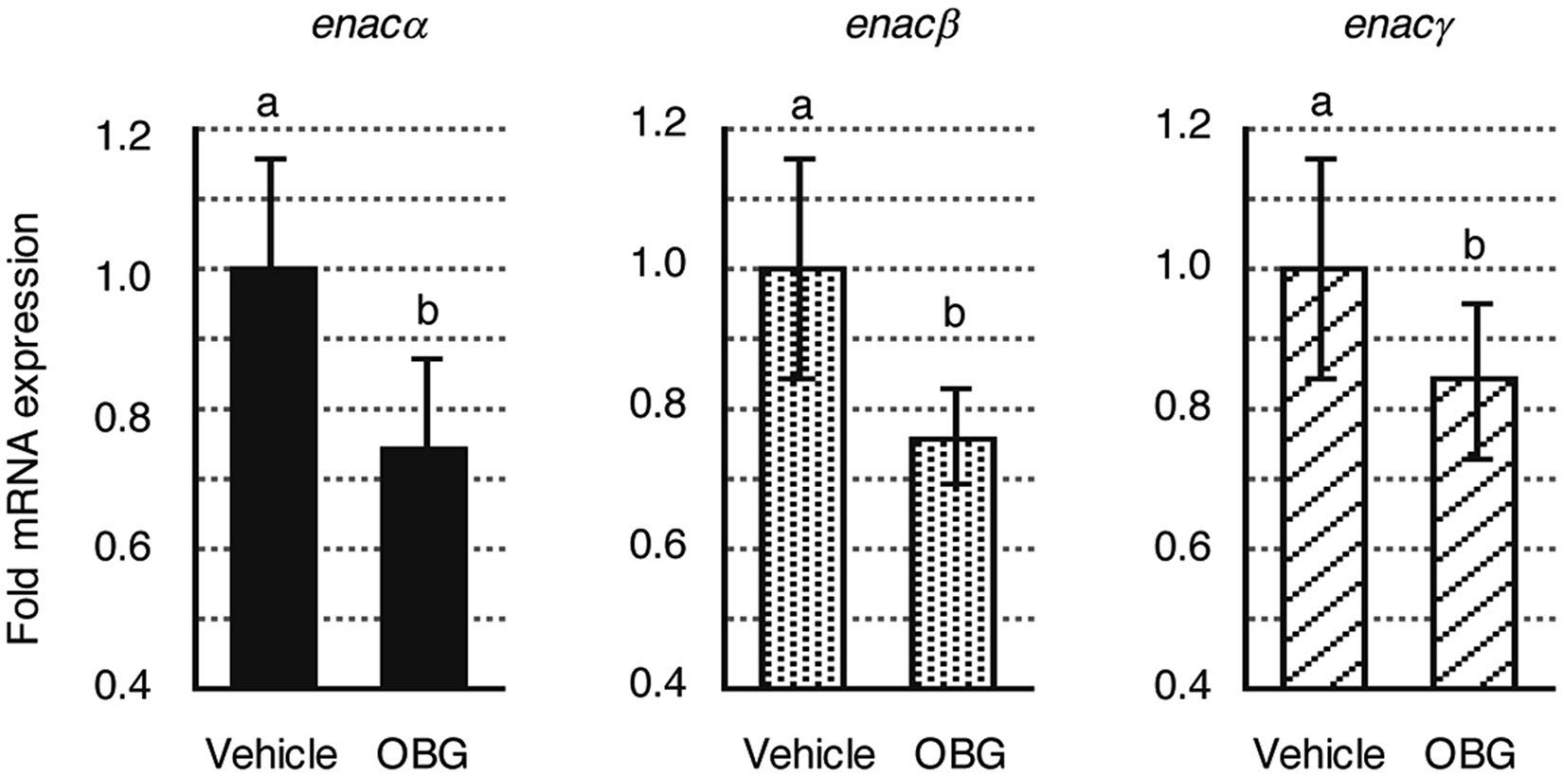
OBG represses *enac* mRNA expression in the mouse kidney. mRNA extracts were prepared from the kidney of mice treated with 1 mg/kg OBG, and the mRNA expression levels of *enac* in extracts were analysed as described in Fig. 4A.

The ENaC-suppressive effect of OBG was further verified *in vivo*. qRT-PCR analysis of *enac* expression was performed in male ddy mouse kidney 6 h after treatment with 1 mg/kg OBG, and OBG significantly down-regulated the mRNA levels of all three *enac* subunits (Fig. 6), without inducing any obvious side effects such as diarrhoea, inflammation or weight loss (data not shown). Thus, we concluded that OBG can suppress the expression of ENaC *in vivo* as well as *in vitro*. The expression level of LXRβ was slightly higher than that of LXRα in the kidney resected from mice compared with M-1 cells (Supplementary Figs S7 and 11).

In conclusion, OBG suppressed ENaC expression in mouse kidney and M-1 cells, presumably by activating LXRβ. OBG therefore appears to be a potent drug lead that does not induce the typical side effects, such as hepatic steatosis, that are observed for conventional LXR ligands such as T0901317 and GW3965.

## Discussion

**Figure 7:**
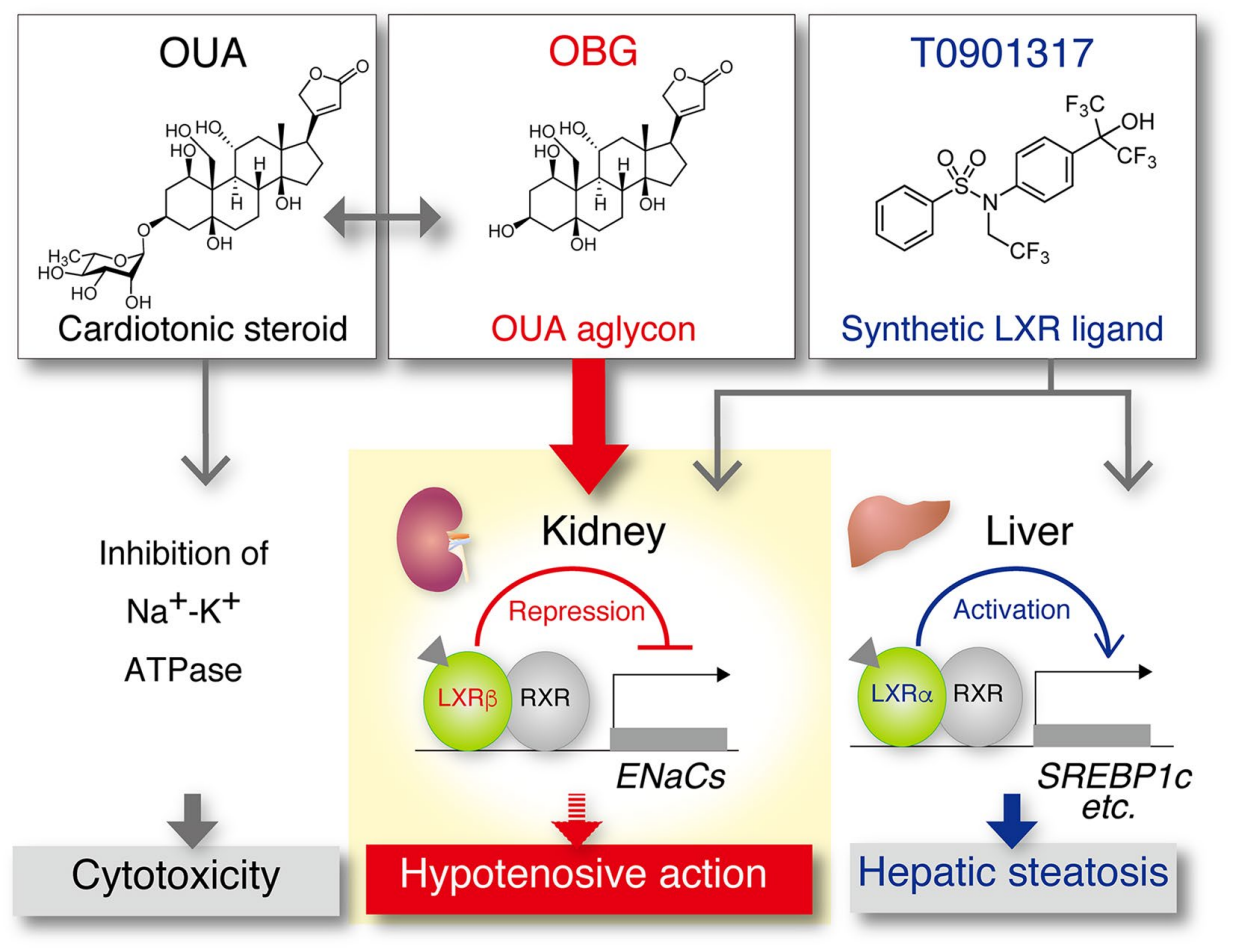
Schematic diagram depicting OBG’s suppressive effect on expression of ENaCs in kidney. OBG, a aglycone of OUA, functions as a LXRβ agonist to down-regulate ENaC in kidney both *in vitro* and *in vivo* where as OUA shows cardiotonic action and cytotoxicity. Notably, OBG doesn’t cause hepatic steatosis which is severe side effect induced by conventional LXR ligands. Thus, OBG is a novel class of LXR ligand as a promising antihypertensive diuretic.

OBG is widely considered to be a biologically inactive biosynthetic precursor of the cardiac glycoside OUA^5,6^. OUA is not only a naturally occurring cardiotonic agent, but also an endogenous digitalis-like factor and potential regulator of blood pressure in mammals^1,2^. Herein, we disclose the importance of OBG as a unique naturally occurring LXR ligand, and demonstrate its potential as a drug lead that does not cause the undesirable side effects that accompany conventional LXR agonists (Fig. 7).

In this study, we screened and identified LXRs among NRs as novel targets of OBG. Based on structural aspects, it is reasonable to speculate that, as a highly oxidised sterol, OBG might modulate LXR signalling, because LXR has a pivotal role as an oxysterol sensor *in vitro* and *in vivo*^27–29,49,50^. Endogenous and synthetic oxysterols activate the diverse biological activities of LXRs in various tissues, and an increased understanding of the subtype- or tissue-specific mechanisms of LXR signalling may assist the development of novel strategies for pharmacological manipulation of LXRs. From this perspective, identification of functional targets of OBG suggests that the kidney is the unexpected pharmacological target tissue, based on LXR transcription, which is surprising since renal oxysterol/LXR signalling has not been previously described.

The kidney maintains extracellular homeostasis by regulating the balance of substances such as sodium and water. In particular, renal reabsorption of Na^+^ is an important part of renal physiology, which is the major determinant of extracellular fluid volume and is consequently thought to affect blood pressure regulation. Potassium-sparing diuretics such as amiloride and triamterene reduce blood pressure independently of renin-based regulation. The collecting duct is the final site of the regulation of urinary Na^+^ transport. In the distal tubule, ENaCs transport Na^+^ ion into epithelial cells, and this is dependent on the electrochemical gradient generated by Na^+^/K^+^-ATPase located on the basal side of the cell. Therefore, a small molecule able to regulate the functional expression of ENaCs in ductal collecting cells could potentially modulate blood pressure through Na^+^ reabsorption. Mineralocorticoids are hypertensive NR ligands that up-regulate ENaC expression during renal regulation of blood pressure. However, the hormone/NR signalling process that functions in the reverse direction is unknown. While it was recently reported that synthetic LXR ligands exert an inhibitory effect on Na^+^ ion transport in collecting duct cells by down-regulating ENaC^22^, the molecular mechanisms responsible, including the LXR dependency, remain uncertain. From this standpoint, our results suggest that OBG/LXR signalling might contribute to the antihypotensive effects by down-regulating ENaC expression. Thus, up-regulation of ENaC in epithelial cells could likely cause hypertension via fluid retention in the blood vessel, whereas down-regulation will cause hypotension by promoting the excretion of fluid via urine.

LXRα is mainly expressed in the liver, kidney, intestine and adipose, as well as macrophages, whereas LXRβ is ubiquitously expressed in mammals^51,52^. This difference in expression pattern in organs suggests that each isoform plays a different role. Indeed, efflux of cholesterol from macrophages, activation of genes related to lipid metabolism and induction of hepatic steatosis are all dependent on LXRα^34,53,54^, whereas the biological role of LXRβ is not clearly understood, despite some speculation^55,56^. Unexpectedly, we found that ENaC was down-regulated by OBG in a LXRβ-specific manner, even though OBG was not selective for LXRβ in reporter assays, competitive binding assay and *in silico* binding assays. To the best of our knowledge, this is the first example of a subtype-specific function of LXRs, although we cannot exclude the possibility that the LXRβ-selective action of OBG is partly attributed to the 100-fold higher expression levels of LXRβ than LXRα in M-1 cells. However, our results showed that *lxr*α overexpression was ineffective at down-regulating *enac* with LXR ligands OBG and T0901317, indicating that an unidentified determinant related to the LXR subtype specificity of OBG may contribute to tissue selectivity, since OBG did not exert agonistic activity in the liver, where LXRα is abundantly expressed. Furthermore, the possibility that metabolism of OBG in the liver resulted in a loss of LXR-binding ability was refuted because OBG did not enhance the mRNA expression levels of genes related to lipogenesis in M-1 cells, unlike T0901317. Thus, the inability of OBG to up-regulate lipogenesis-related genes revealed that its action is not liver-specific. We hypothesise that an unknown mechanism accounts for the tissue-specific activation of LXR by OBG.

Animal experiments fortuitously revealed unique features and several advantages of OBG versus conventional LXR agonists T0901317 and GW3965. To date, the positive effects of pan-LXR synthetic ligands have been coupled with an undesirable increase in serum and hepatic triglyceride levels, probably through LXRα action in the liver, leading to hepatic steatosis. Thus, the development of tissue-selective LXR ligands is highly desirable from a pharmacological standpoint. Our results reveal for the first time that LXR could be targeted by naturally occurring substances such as OBG without the undesirable side effects associated with conventional LXR ligands.

Unlike T0901317, OBG did not cause hepatic steatosis in mice nor up-regulate the genes associated with lipid metabolism in human hepatocyte (Fig. 2A–E). This can be attributed to the high LXR-selective agonistic activity of OBG and the absence of cross-reactivity with other NRs, because T0901317 also functions as a ligand of FXR, a reported master regulator of hepatic triglycerides^57^. In addition to the low cytotoxicity and inability to induce G1 cell cycle arrest, this high LXR selectivity confirms OBG as a member of a unique class of LXR ligands. Future work could identify an even better LXR ligand, and could also reveal why OBG does not include hepatic steatosis despite its agonistic activity towards LXRα.

Our results showed that OBG suppressed the expression of ENaC-β and -γ in M-1 cells, but not the α subunit, whereas all three subunits of ENaC were down-regulated at the mRNA level in the mouse kidney following administration of OBG. Taking that the *enac*-α repressive effect by 10^−5^ M T0901317 in M-1 cells was previously reported by Soodvilai *et al*.^22^, only for *enac-*α, the unveiled mechanisms related in the ligand binding may be underlying, since the treatment of T0901317 at the concentration of 10^−6^~10^−8^ M could not effect *enac-*α expression in our more than ten times trial.

ENaC-β and -γ are encoded by genes that are in close proximity on the chromosome (Chr 16 in human, Chr 7 in mouse), while the α subunit is located elsewhere (Chr 12 in human, Chr 6 in mouse). Thus, the same LXRβ-dependent trans-repressive activity of OBG might influence the expression of β and γ subunits. Indeed, one aspect of the trans-repressive function of LXR has been described^58^. The authors showed that LXR SUMOylation is induced by LXR agonist binding, and SUMOylated LXR hinders the clearance of co-repressors, leading to inhibition of the transcriptional activation of pro-inflammatory genes stimulated by NF-κB^59^ or lipopolysaccharides (LPS)^60^. We therefore raise the possibility that OBG-bound LXR exerts trans-repression of ENaC expression, and further studies are in progress to test this hypothesis.

Alternatively, OBG-bound LXRs might adopt a unique structure distinct from LXRs bound to conventional ligands. Indeed, ligand-induced structural alterations of NRs are known to generate diversity in the interaction of co-regulators with NRs^13–16^. In this study, because OBG was found to exert biological effects different from those of other known LXR ligands, it is likely that OBG-bound LXR directly interacts or associates with a particular set of co-regulators, at least in the liver and kidney. In addition, OBG was unable to exert agonistic activity for mouse LXRs tagged with Myc-DDK at C-termini, while T0901317 could activates the mouse LXRs as well as human LXRs. These results suggest the possibility that OBG induces the unique allosteric conformational changes in the ligand binding domain in a different mechanism from T0901317, because it is known that NR’s tag at C-termini sometimes could affects the NR/co-factor interactions (Supplementary Fig. S12). The whole concept of co-regulators supporting and facilitating the functions of NRs such as LXR awaits further study. It would therefore also be interesting to identify a co-regulator(s) that accounts for the unique features of OBG as a naturally occurring LXR ligand.

Although to the best of our knowledge the pharmacokinetics of OBG haven’t been reported, that of digoxin^61^ and bufalin^62^ both are members of cardiotonic steroids were investigated. Digoxin was deglycosylated in the liver and resultant aglycone was conjugated as it was or after epimerization at C-3. Bufalin was also metabolized in the liver to be 3-keto and 3-epi congeners as inactive form. The concentration of bufalin, non-glycoside, in blood after *po* administration was raised faster (T_max_: 15 min), then fallen faster (T_1/2_: 2.6 h) than that of digoxin, triglycoside (T_max_: 1 h, T_1/2_: 3.6 h). Bufalin was excreted in the bile (75%) and in the urine (1.3%). Taking pharmacokinetic property of these cardiotonic steroids into account, OBG, non-glycoside, could be expected to be also readily absorbed, then metabolized in the liver to be inactive 3-epimer which would be excreted primarily in bile like bufalin.

In summary, we revealed that OBG functions as a LXR-selective agonist and suppresses the expression of ENaC subunits in kidney collecting duct cells and the mouse kidney via a LXRβ-mediated pathway. Down-regulation of ENaC in the kidney presumably decreases Na^+^ ion transport and water resorption, leading to antihypertension. Moreover, we characterised several unique features of OBG in hepatic steatosis and cellular events. In addition, OBG didn’t derive any obvious toxic damages to mice such as diarrhoea, inflammation or weight loss in continuous five days treatment. Thus, OBG holds promise for the development of a potent antihypertensive diuretic drug without the undesirable side effects of conventional LXR ligands.

## Methods

### Chemicals

OUA and T0901317 were purchased from (Sigma), whereas GW3965 and 22R-hydroxycholesterol were from (Sigma Aldrich) and (Cayman Chemical Co.), respectively. OBG was synthesised from OUA by de-glycosylation using naringinase followed by column chromatographic purification^63^. MeOH, EtOH, DMSO and carboxymethylcellulose were purchased from (Wako Pure Industries, Ltd).

### Luciferase assay

Human embryonic kidney 293 T cells were seeded on 12-well plates at a density of 1.5 × 10^5^ cells/well in Dolbecco’s Modified Eagle Medium (DMEM) (without phenol-red) supplemented with 10% charcoal-stripped Fetal Bovine Serum (FBS) (Biological Industries Ltd.). After 24 h, cells were transfected using lipofectamine 2000 (Life Technologies Corp.) with 25 ng of each NR expression plasmid, 250 ng of each NRE-luciferase reporter plasmid and 1 ng of pCMV-Renilla luciferase reporter plasmid (controls) in 400 μL of OptiMEM (Life Technologies Corp.) per well. For LXR, VDR and FXR, 25 ng of plasmid encoding RXR was also added. After a 6 h incubation, the supernatant was removed and 1 mL of DMEM (without phenol-red) supplemented with 10% charcoal-filtered FBS and 1 μL of sample in EtOH solution were added. After another 48 h incubation, cells were washed with D-PBS (-) and lysed, and luciferase activity was measured with the Dual-Luciferase Reporter Assay System (Promega) on a GloMax 96 Microplate Luminometer (Promega). Firefly luciferase activity was normalised against *Renilla* as an estimate of the activation of NRs.

### Competitive binding assay using fluorescent anisotropy

The fluorescent LXR ligand was synthesized according to the reported procedure^24^. *N*-Terminal GST tagged LXRα and β LBD was expressed in *E*. *coli*. strain BL211(DE3) cultivated in 2 × YT medium. Expression of the recombinant protein was induced by the addition of 0.1 mM isopropyl-β-D-thiogalactoside at OD_600_ = 1.0. After 5 h induction at 20 °C, the cells were harvested by centrifugation (5000 rpm, 5 min) and the resulted cell pellet was washed with PBS then re-suspended in lysis buffer (PBS contained 1% Triton X-100, protease inhibitor cocktail). After disruption of the cells by sonication, the supernatant was incubated with Glutathione Sepharose 4B for 1 h at 4 °C. After the resin was washed with PBS, the LBD was eluted with buffer (10 mM HEPES, 150 mM NaCl, 2 mM MgCl_2_, 5 mM DTT, 10 mM glutathione, pH 7.9). Each concentration of eluted LBD was quantified by Bradford assay, and the purity was determined by sodium dodecyl sulfate polyacrylamide gel electrophoresis (SDS-PAGE). Fluorescent anisotropy was detected on a JASCO FP-6500 Spectrofluorometer at λex = 400 nm and λem = 500 nm. Fluorescent LXR ligand (0.5 μM) and LXRα and β-LBD (0.5 μM) were incubated in the buffer (10 mM HEPES, 150 mM NaCl, 2 mM MgCl_2_, 5 mM DTT, pH 7.9), then OBG or T0901317 was added to this solution.

### *In silico* docking

All docking simulations were performed using AutoDockTools (version 1.5.7rc1, Molecular Graphics Laboratory, The Scripps Research Institute) running on Windows 7 Professional. Firstly, the reported co-crystal structures of LXR and T0901317 (PDB code 1UHL for LXRα and 1PQC for LXRβ) were downloaded as templates, and T0901317 was replaced with the other ligand and the conformation was minimised by MM2 calculation. The LXR-ligand complexes were subjected to docking simulations, and the stabilisation energy was calculated.

For the VDR and FXR, VDR and 1α, 25-dihydroxyvitamin D3 (PDB code: 1DB1), and FXR and fexaramine (PDB code: 1OSH), were used as templates, and subsequent procedures were the same as described above for LXR.

### Animal experiments

All animal experiments were approved by the ethics committee of Tohoku University and carried out in accordance with the relevant guidelines.

#### *In vivo* testing of the suppressive effect on ENaCs

After housing, male ddy mice were divided into two groups at random; vehicle (n = 5) and 1 mg/kg OBG (n = 4). Each sample was suspended in 0.5% aq. carboxymethylcellulose and administered intraperitoneally (0.1 mL/10 g). Both kidneys were removed under anaesthesia by pentobarbital at 6 h after administration, sliced immediately into 2 mm sections, frozen in liquid nitrogen and stored at −80 °C until needed.

#### *In vivo* testing for steatosis in liver

After housing, male C67BL/6 mice were divided into three groups at random: vehicle (n = 5), 10 mg/kg OBG (n = 4) and 10 mg/kg T0901317 (n = 4). Samples were suspended in 0.5% aq. carboxymethylcellulose and DMSO (ratio = 2:1) and administered intraperitoneally (0.15 mL/10 g) once a day for 5 days. On the day after the final administration, the liver was removed under anaesthesia by pentobarbital, frozen in liquid nitrogen and stored at −80 °C until needed. Triglyceride analysis was performed as previously described^21^. Briefly, ~100 mg of liver (wet weight) was homogenised in 0.5 mL of MeOH using a Biomasher (Nippi Inc.). The homogenised suspension was transferred to a new tube, and another 0.5 mL of MeOH was added and homogenisation was repeated five times. The entire 3 mL MeOH extract was mixed with 6 mL of CHCl_3_ and incubated at RT for 30 min. The supernatant (2 mL) was dried under an N_2_ stream at 40 °C, and the resultant residue was dissolved in 0.5 mL of 50% aq. DMSO. The concentration of triglycerides was measured using a Triglyceride E-test Wako kit (Wako Pure Industries, Ltd.) in accordance with the instructions.

### Cell lines

Human embryonic kidney cell line 293 T (CRL-3216, ATCC), murine hepatocytes Hepa1–6 (CRL-1830, ATCC) and human hepatocyte carcinoma HepG2 (HB-8065, ATCC) were maintained in Dulbecco’s minimum essential medium (DMEM) supplemented with 10% fetal bovine serum (FBS). Murine collecting duct cell line M-1 (CRL-2038, ATCC) was maintained in DMEM/Ham’s F-12 media supplemented 5% FBS and 5 μM dexamethasone. Human colorectal carcinoma cell line HCT116 (CCL-247, ATCC) was maintained in RPMI 1640 with L-glutamine containing 10% FBS, which was replaced with the medium containing 1% FBS. All cell lines were cultivated at 37 °C in a humidified 5% CO_2_ atmosphere environment.

### Plasmids

For luciferase assay, the NR plasmids (pSG5-hLXRα, pUC-hLXRβ, pcDNA3-hAR, hPR-A and hPRB, pcDNA3-Flag-tagged hRXR, hVDR hFXR and hERα, pcDNA5/FRT/TO-Flag-tagged hERβ) and their cognate NRE-Luciferase reporter plasmids (pGL3-tk-DR3, DR4 and PRE/ARE, pGL3-SHP and E1b) were kindly gifted by Kato S. The mouse LXRα and LXRβ plasmids (pCMV-entry-mLXRα (MR207128) or mLXRβ (MR227522) tagged with Myc-DDK at C-termini) were purchased from (Origene).

### Real time-qPCR analysis

Murine collecting duct M-1 cells were seeded on 12-well plate at 1.5 × 10^5^ cells/well in the cultivation medium and incubated for 24 h. The corresponding concentration of sample solution in EtOH 1 μL was added, then incubated for another 24 h. Total RNA was isolated using RNeasy Plus Mini Kits (QIAGEN) according to manufacture’s protocol. cDNA synthesis was performed using ReverTra Ace (TOYOBO Co. Ltd.) with oligo-dT at 42 °C for 30 min. Quantitative PCR amplification was performed using KAPA SYBR FAST ABI Prism qPCR lit (KAPA Biosystems) and the STEP ONE (GE Healthcare). PCR reactions were carried out for 40 cycles at 95 °C for 5 s at 60 °C for 30 s and the data were analyzed with comparative C_T_ method using glyceraldehyde 3-phosphate dehydrogenase (GAPDH) as an internal control. PCR products were subjected to melting curve analysis and separated by electrophoresis in 2% agarose gels followed by staining with ethidium bromide in order to ensure single DNA duplexes.

The sequences of specific primers were as follows.

*gapdh*; forward: 5′-CATGGCCTCCAAGGAGTAAG-3′

reverse: 5′-GAGGGAGATGCTCAGTGTTG-3′

*lxr-*α; forward: 5′-CCCATGGACACCTACATGC-3′

reverse: 5′-GCTTCAGTTTCTTCAAGCGG-3′

*lxr-*β; forward: 5′-AGGTCTTTGCATTGCGACTC-3′

reverse: 5′-CATCTTCAAGAAGACACCACC-3′

*enac-*α; forward: 5′-GGCTCTTCTGCCTGTGCT-3′

reverse: 5′-GGAAGATGTGCTGAAGTGAC-3′

*enac-*β; forward: 5′-GTCGGACAGTGAGGTGGAG-3′

reverse: 5′-CAAGGCCTAATGAAACGAGG-3′

*enac-*γ; forward: 5′-CTTGGACAGTGCCTTTTCCT-3′

reverse: 5′-GAAGAGAGTCTCCTCAAACC-3′.

*abca1*; forward: 5′-CCTGCGTTTCTGTGGAGAAG-3′

reverse: 5′-GCCCCTGTAATGGAATTGTG-3′.

*srebp1c*; forward: 5′-GACCTTTGTCATTGGCTGTG-3′

reverse: 5′-GCCTCTGCAATTTCCAGATC-3′.

*fas*; forward: 5′-CGTAGTGGGGTTCCCAGAG-3′

reverse: 5′-CGGTAGAAAAGGCTCAGTTTG-3′.

The same procedure was applied to the evaluation of mRNA level in human cell lines. The sequences of specific primers were as follows.

*GAPDH*; forward: 5′-ATGAGTCCTTCCACGATACC-3′

reverse: 5′-ATCCCATCACCATCTTCCAG-3′

*LXR-*α; forward: 5′-GGCTGCCTCCTAGAAGTGG-3′

reverse: 5′-CTCTTTTAATGCCACGGGAG-3′

*LXR-*β; forward: 5′-GACCACCCTCCAGCAGATAG-3′

reverse: 5′-CATCTTCAAGAAGACACCACC-3′

*ENAC-*α; forward: 5′-CATCTCCAGGGGGCTCT-3′

reverse:5′-GATGAGAGCCGATAGGTCTA-3′

*ENAC-*β; forward: 5′-CACTGAGCAGCCAAGACTGT-3′

reverse: 5′-CTCTGTTGAAGGACACAAGC-3′

*ENAC-*γ; forward: 5′-CCAGCTCACAGATACCCAGA-3′

reverse: 5′-GATCCATGTCCTTAGACCATG-3′.

*ENAC-*δ; forward: 5′-ACCAGCAGCCCAGGAAG-3′

reverse: 5′-GTGTAGGTGGTTTGACCAGAG-3′.

*ABCA1*; forward: 5′-GTGGACGTTGCAGTTCTCAC-3′

reverse: 5′-CACAACACTTCACATGGTGC-3′.

*SREBP1C*; forward: 5′-CATGATGGTGCTGACCTCTG-3′

reverse: 5′-GGAAATGTACCCCTCTCTTC-3′.

*FAS*; forward: 5′-CCTGCACAGGCACACAGG-3′

reverse: 5′-CCAAACATGGAGTTGGTGCC-3′.

### siRNA transfection

M-1 cells were seeded on 12-well plate at 1.0 × 10^5^ cells/well in the cultivation medium and incubated for 24 h. After removal of supernatant, the mixture of DharmaFECT 0.8 μL and siRNA 80 pmol in OptiMEM 400 μL was added for each well. The mixture was prepared in accordance with manufacture’s instruction of DharmaFECT. After another 24 h incubation, supernatant was replaced with cultivation medium with corresponding concentration of sample solution in EtOH (final concentration of EtOH is 0.1%), then further 24 h incubated. Subsequent procedure was same as evaluation of expression level of mRNA. In case of LXRα over-expression and LXRβ knock down condition, M-1 cells were exposed to LXRβ knock down medium same as above for 1 d, then LXRα over-expression medium by use of 200 ng/well of LXRα of plasmid for 6 h. After that, M-1 cells were treated in the same manner as evaluation of the expression level of ENaC.

The sequence specific siRNA pairs were as follows.

*lxr-α*:

sense: 5′-UCUUCAAGCGGAUCUGUUCUU-3′

antisense: 5′-GAACAGAUCCGCUUGAAGAAA -3′

*lxr-β*:

sense: 5′-CCAGCCUUGGUGGUGUCUUCUUGAA-3′

antisense: 5′-UUCAAGAAGACACCACCAAGGCUGG-3′.

The same procedure was applied to the evaluation of mRNA level in human embryonic kidney 293 T cells. The sequence specific siRNA pairs were as follows.

*LXR-α*:

sense: 5′-UUUAGCAAAGUCAACUAUCUC-3′

antisense: 5′-GAUAGUUGACUUUGCUAAACA-3′

*LXR-β*:

sense: 5′-UUUACAGUGGGUGAAGAAGAA-3′

antisense: 5′-CUUCUUCACCCACUGUAAAGG-3′.

### Cell cycle analysis

The procedure was basically followed that in previous report^39^. In 6-well microculture plates, HCT-116 cells (50 × 10^5^ cells/mL) were cultured in RPMI1640 in the presence of the test samples for 24 h. After whole was washed with D-PBS (-) containing 0.5% BSA and 0.05% NaN_3_ twice, the cells were harvested with trypsin-EDTA solution (Sigma) and then fixed in 70% ice-cold ethanol. DNA was stained with 10 μg/mL propidium iodide in phosphate-buffered saline (D-PBS (-)) for 30 min before analysis on a BD FACSCalibur. Absolute number of viable cells and percent viable cells of total cell population after 24 h incubation with 5 μM GW3965 or OBG in 1% serum was analysed by trypan blue exclusion according to manufacturer’s protocol.

### MTT assay

The cell viability was determined using 3-(4,5-dimethyl-thiazole-2-yl)-2,5-diphenyl tetrazolium bromide (MTT) assay. M-1 cells were seeded on 48 well plates at 2.0 × 10^4^ cells/0.2 mL/well in cultivation medium, then incubated with corresponding concentration of LXR ligands or colchicine (positive control) as EtOH solution (final 0.1% EtOH concentration) for 24 h. At the end of incubation, cells were washed three times with D-PBS (-). MTT reagent was added to each well and cells were incubated for another 4 h at 37 °C. Then, MTT medium was removed and replaced with 150 μL of DMSO. Cell viability was examined by measuring the absorbance at 540 nm with Infinite M200Pro (TECAN). The results of cytotoxicity are expressed as percentage based on the control value as 100% survival.

### Statistical analysis

The results were expressed as the mean ± SD. Statistical analysis was performed using one-way analysis of variance (ANOVA) followed by SNK test. A *p* value below 0.01 (*p* < 0.01) was considered to be significantly different.

## Electronic supplementary material

Supplementary information
