# Supplementary material for: Ouabagenin is a naturally occurring LXR ligand without causing hepatic steatosis as a side effect
Source: Sci Rep. 2018 Feb 2;8:2305. doi: 10.1038/s41598-018-20663-z (PMC5797171; doi:10.1038/s41598-018-20663-z)
Supplement: Supplementary file 1 — Supplementary information [file 41598_2018_20663_MOESM1_ESM.pdf]

SUPPLEMENTARY INFORMATION for

Ouabagenin is a naturally occurring LXR ligand without causing hepatic steatosis as a side effect.

Satoru TAMURA,[1,7,9] Maiko OKADA,[2,8,9] Shigeaki KATO,[3,4] Yasuharu SHINODA,[5] Norifumi SHIODA,[5] Kohji FUKUNAGA,[5] Kumiko TEI[6], and Minoru UEDA[1,10,*]

[1] Department of Chemistry, Graduate School of Science, Tohoku University, Sendai, Miyagi, 980-8578, Japan.

[2] Institute of Medical Science, St. Marianna University Graduate School of Medicine, Kawasaki, Kanagawa, 970-8551, Japan.

[3] Iwaki Meisei University, Iwaki, Fukushima, 970-8551, Japan.

[4] Research Institute of Innovative Medicine, Tokiwa Foundation, Iwaki, Fukushima, 972-8322, Japan.

[5] Graduate School of Pharmaceutical Sciences, Tohoku University, Sendai, Miyagi, 980-8578, Japan

[6] Graduate School of Science, The University of Tokyo, Tokyo, 113-0032, Japan

[7] Present address: School of Pharmacy, Iwate Medical University, Shiwa-gun, Iwate, 028-3694, Japan.

[8] Present address: Genome regulation and Molecular Pharmacogenomics, School of Bioscience and Biotechnology, Tokyo University of Technology, Hachioji, Tokyo, 192-0982, Japan.

[9] These authors contributed equally to this article.

[10] Lead Contact

Supplementary Figures and Table

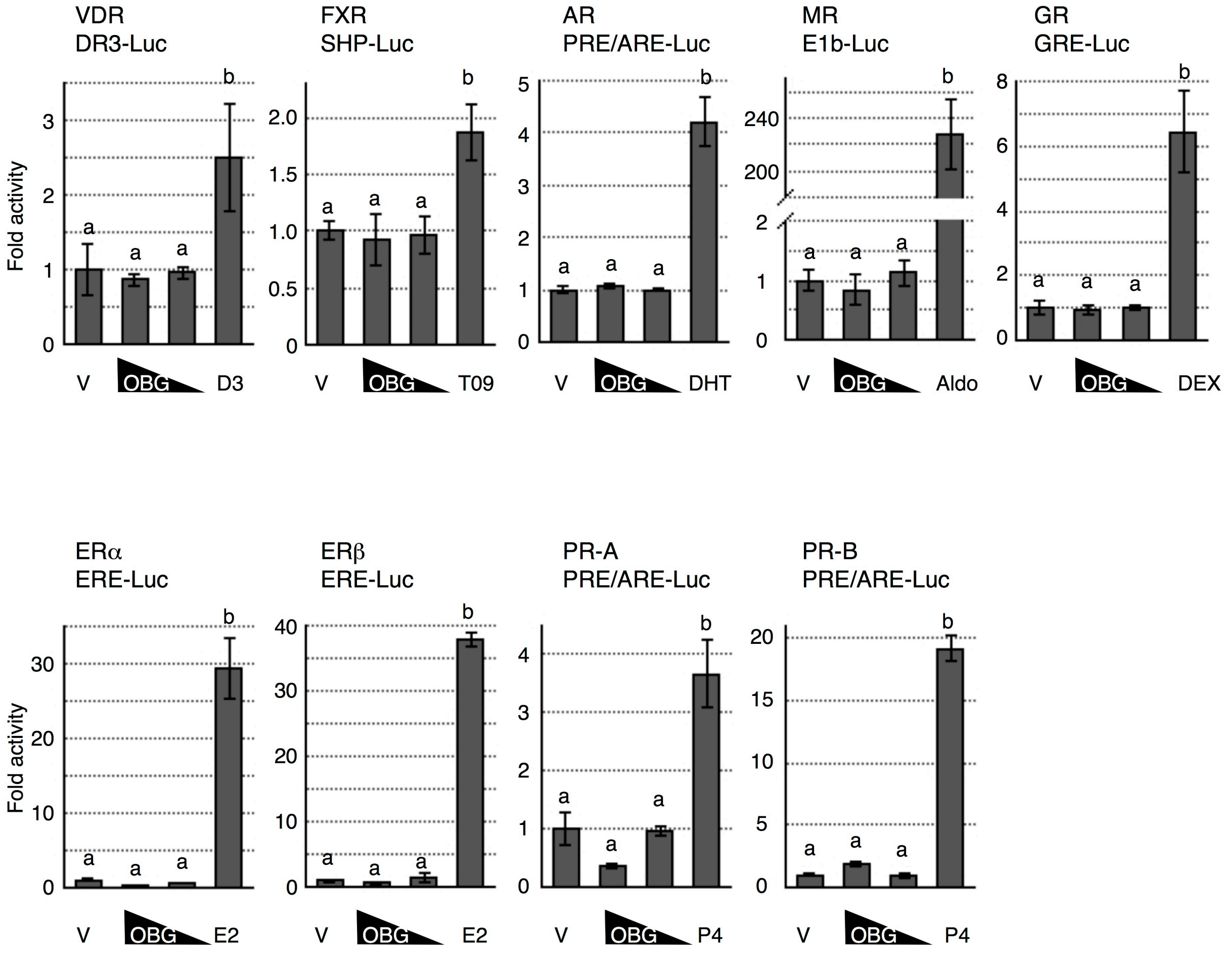

Fig. S1. OBG did not remarkably affect the transcriptional activities of many NRs.

OBG was subjected to luciferase reporter assay with many NRs and its cognate reporter plasmids in 293T cell lines. The transcriptional activity of steroid receptors (AR, ER, PR, GR, MR), VDR and FXR were not affected by OBG. 10^{-8} M or 10^{-9} M OBG and 10^{-8} M corresponding NR ligands were treated 48h before the assay. Each luciferase activity of OBG was normalized by renilla luciferase activity (as a control), comparing with that of vehicle. Data are represented as mean ± SD and the values followed by different letters are statistically different according to analysis of variance followed by SNK test ($P < 0.01$). V: vehicle, D3: vitamin D3, T09: T0901317, DHT: dehydrotestosterone, Aldo: aldosterone, DEX: dexamethasone, E2: estradiol, P4: progesterone.

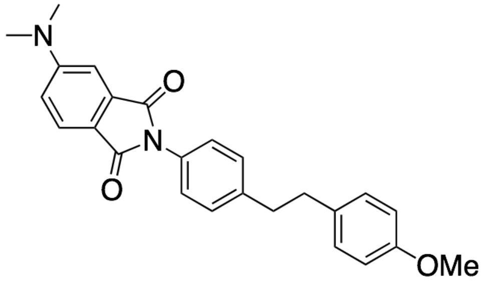

Fig. S2. Chemical structure of known fluorescent LXR ligand

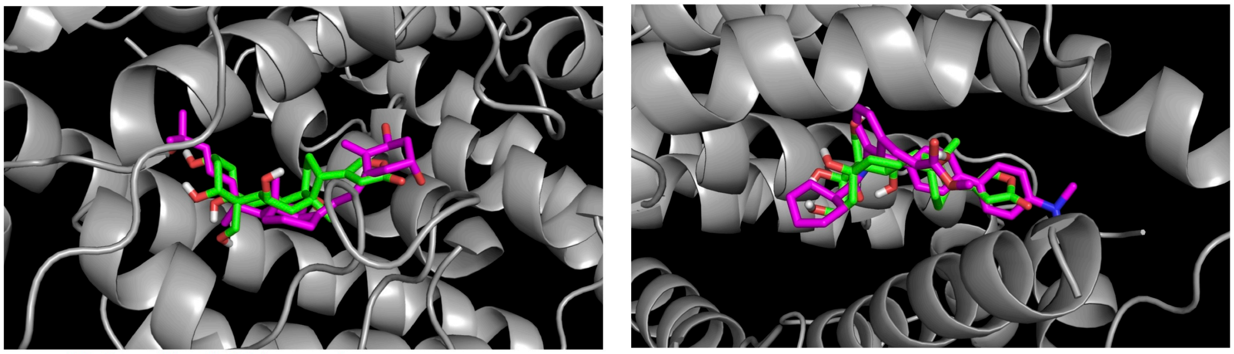

VDR with OBG (green) or
1α,25-dihydroxyvitamine D3 (magenta)

FXR with OBG (green) or fexaramine (magenta)

Fig. S3. Docking simulation of VDR or FXR with their ligands.

Using the previous reported data sets of the crystal structure for VDR/1α,25-dihydroxyvitamin D3 complex and FXR/fexaramine complex, OBG was subjected to *in silico* docking simulation and compared with corresponding known ligands. In left panel, gray ribbon; VDR, green; OBG, magenta; 1α,25-dihydroxyvitamine D3. In right panel, gray ribbon; FXR, green; OBG, magenta; fexaramine.

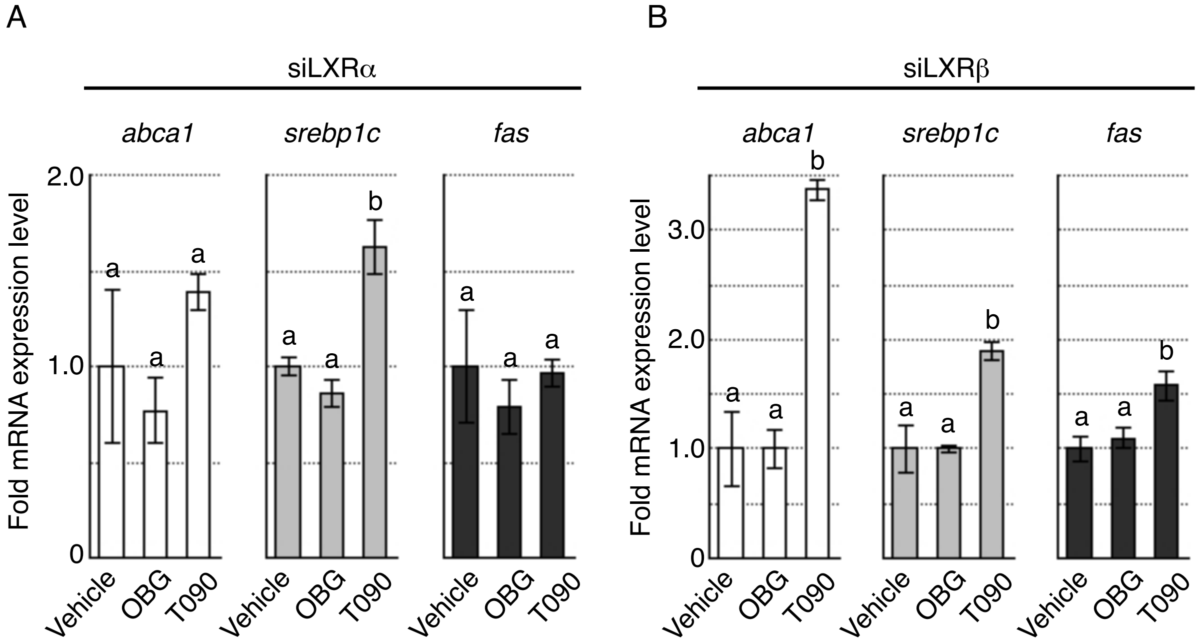

Fig. S4. Up-regulation of *abca1* and *fas* by T0901317 was cancelled under LXRα knockdown.

mRNA expression levels of *abca1*, *srebp1c* and *fas* in M-1 cells after treatment with LXR ligands under LXRα (A) or LXRβ (B) knockdown conditions. The expression levels of lipogenesis-related genes were evaluated, one day after treatment with each ligand at 10^{-6} M concentration under LXR knockdown condition. The amounts of mRNA were normalized by that of *gapdh*, then compared with that of vehicle. The data are represented as mean ± SD and the values followed by different letters are statistically different according to analysis of variance followed by SNK test ($P < 0.01$).

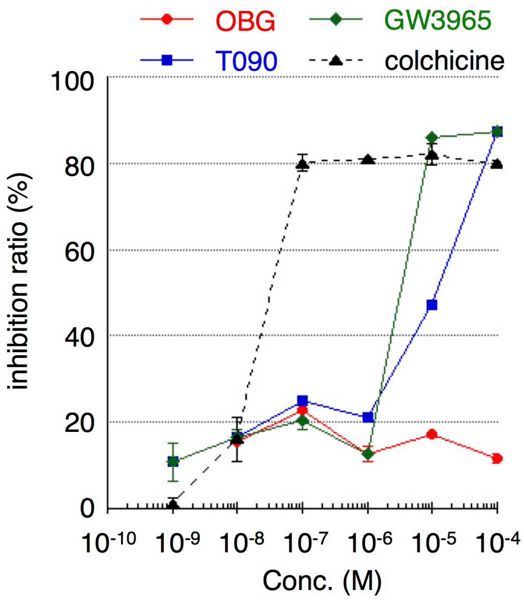

Fig. S5. OBG caused little inhibition of M-cell proliferation.

Cytotoxicity test by MTT assay for M-1 cells. After treatment with each test sample for 24 h, M-1 cells were subjected to MTT assay in order to evaluate their viability. The inhibition ratios are expressed as percentage based on the viability of control condition as 100%. Red: OBG, blue: T0901317, green: GW3965, black: colchicine (positive control). The data are represented as mean ± SD.

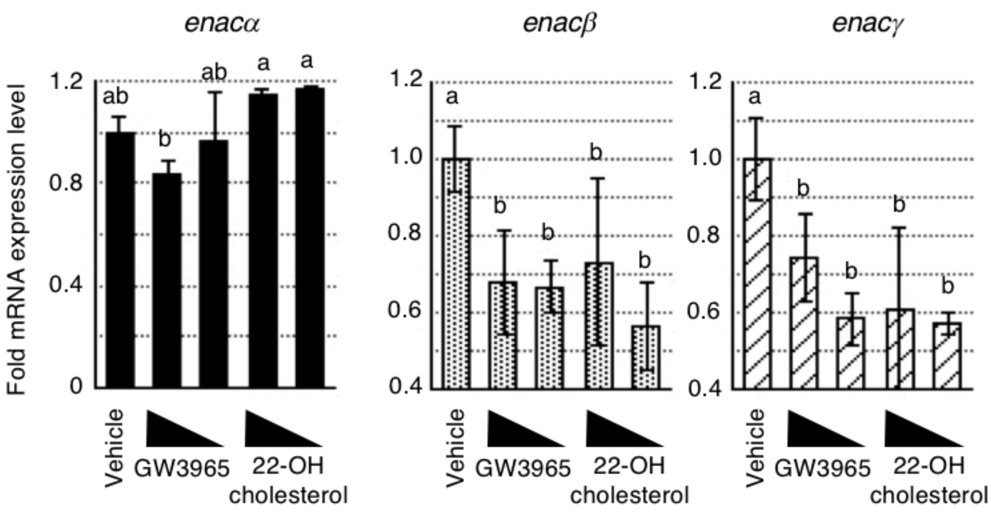

Fig. S6. Conventional LXR ligands decrease mRNA levels of *enac-β and -γ* in M-1.

mRNA levels of each subunit of *enac* in M-1 cells after treatment with LXR ligands. After treatment with each ligand at 10^{-6} M or 10^{-7} M concentration for 1 d, the expression level of *enacs* were evaluated. The amounts of mRNA were normalized by that of gapdh, then compared with that of vehicle. The data are represented as mean ± SD and the values followed by different letters are statistically different according to analysis of variance followed by SNK test ($P < 0.01$). 22-OH cholesterol means 22-hydroxycholesterol.

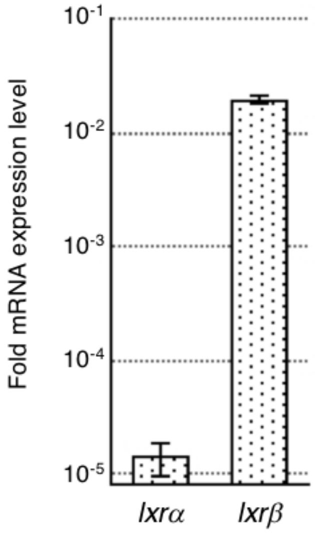

Fig. S7. The mRNA level of *lxrα* in M-1 cells was less than 100 times that of *lxrβ*.

The mRNA level between LXRα and β in M-1 cells was compared by qRT-PCR. The data were normalized by that of *gapdh* and represented as mean ± SD.

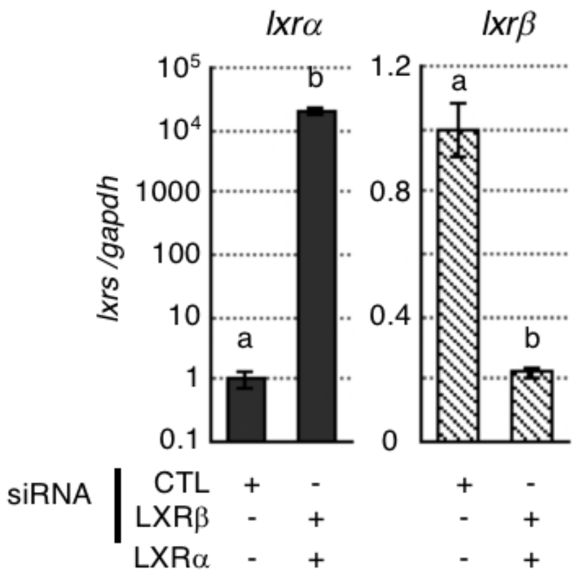

Fig. S8. The confirmation of the efficiency for the over-expression efficiency of LXRα in LXRβ knocked down M-1 cells.

The M-1 cells were treated with LXRα plasmid for 6 h after treatment with siRNA for LXRβ for 24 h. The expression level of *lxr*s was evaluated by real time-qPCR analysis in the same manner as Fig. 5A. The data were normalized by that of *gapdh* then compared with that of intact. The graphs are represented as mean ± SD and the values followed by different letters are statistically different according to analysis of variance followed by SNK test ($P < 0.01$).

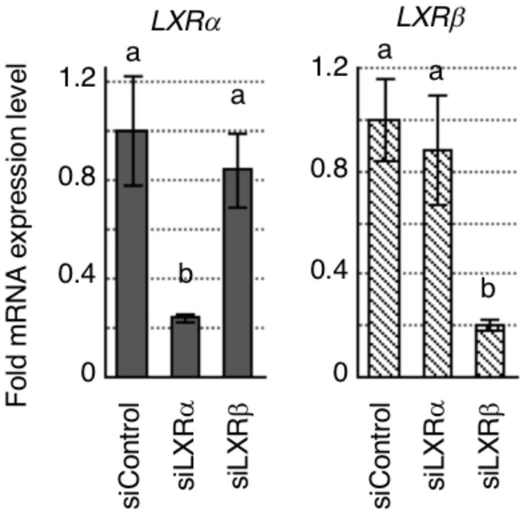

Fig. S9. LXR subtype-selective knockdown in 293T cells

LXRα or β subtype-selective knockdown in M-1 cells was performed by transfection of corresponding siRNAs. The efficacy and selectivity were confirmed by qRT-PCR. The amount of mRNA was normalized against *GAPDH*, then compared with siControl.

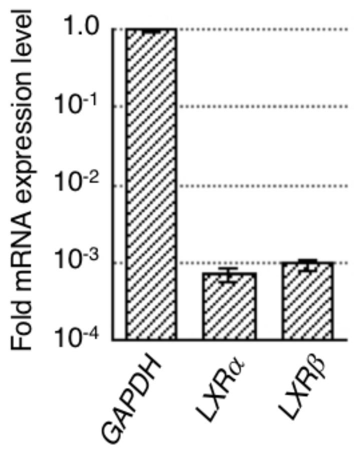

Fig. S10. mRNA expression level of both subtype of LXR in 293T cells were almost same.

The mRNA level between LXRα and β in 293T cells was compared by qRT-PCR. The data were normalized by that of *gapdh* and represented as mean ± SD.

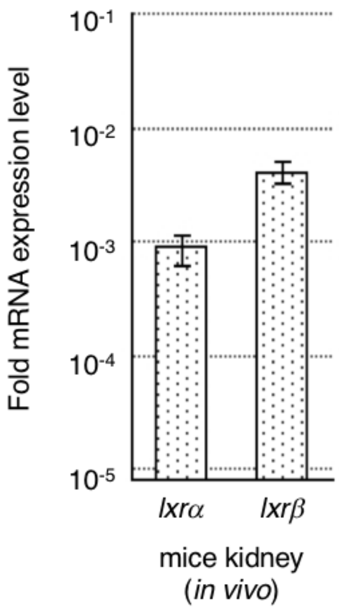

Fig. S11. mRNA expression level of LXRβ was a little more than that of LXRα in mouse kidney.

The mRNA level between LXRα and β in mouse kidney was compared by qRT-PCR. The data were normalized by that of *gapdh* and represented as mean ± SD.

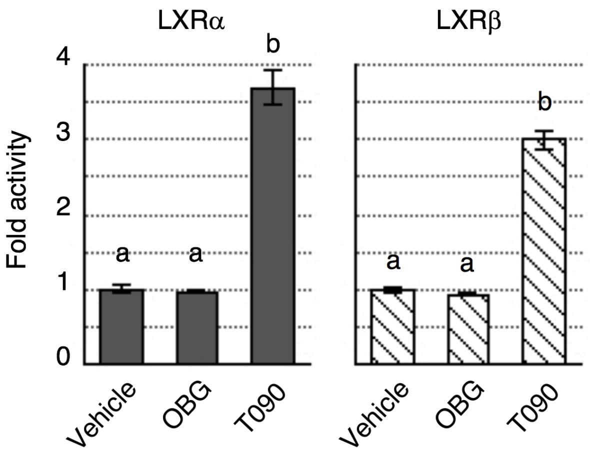

Fig. S12. OBG but not T0901317 agonistic activity towards C-terminal tagged-mouse LXRs could not be observed in luciferase reporter assay,

The expression vector for mouse LXRα or LXRβ C-terminal tagged with Myc-DDK was subjected to luciferase reporter assay in 293T cells as described in Fig. S1. The fold change in transcriptional activity is expressed as the mean ± SD of triplicate experiments. Values followed by different letters are statistically different according to analysis of variance (ANOVA) followed by SNK tests ($P < 0.01$).

Table S1. Stabilization energies of complex of MR, GR or AR with ligands in docking simulation.

The table displays the stabilization energy calculated based on the resultant complex structure of receptor and ligand by *in silico* docking study.

Ligands	MR	GR	AR
OBG	-6.3	-8.8	-6.6
aldosterone	-12.3	-	-
DEX	-	-12.3	-
testosterone	-	-	-11.4

(kcal/mol)

Table S2. Stabilization energies of complex of VDR or FXR with ligands in docking simulation.

The table displays the stabilization energy calculated based on the resultant complex structure of receptor and ligand by *in silico* docking study in Fig. S3.

Ligands	VDR	FXR
1α,25-dihydroxyvitamin D3	-12.2	-
OBG	-8.7	-7.0
fexaramine	-	-11.3

(kcal/mol)
